# A Clinical Randomized Controlled Trial of Acupuncture Treatment of Gastroparesis Using Different Acupoints

**DOI:** 10.1155/2020/8751958

**Published:** 2020-04-25

**Authors:** Wu Xuefen, Li Ping, Liu Li, Chen Xiaoli, Zenghui Yue

**Affiliations:** College of Acu-Moxibustion and Tuina, Hunan University of Traditional Chinese Medicine, Changsha, Hunan 410208, China

## Abstract

**Objective:**

To explore the effect of “selecting acupoints by site” on the synergy effect of “acupoint compatibility” according to the clinical efficacy of acupuncture treatment of patients with gastroparesis.

**Methods:**

A total of 99 patients who met the diagnostic criteria for gastroparesis were enrolled in 3 clinical centers and randomly divided into group A (33 cases), group B (33 cases, 1 case of shedding), and group C (33 cases, 1 case of shedding). In group A, acupuncture was performed at Zhongwan (CV 12) and Zusanli (ST 36); in group B, acupuncture was performed at Neiguan (PC 6) and Zusanli (ST 36); in group C, acupuncture was performed at nonacupoint and Zusanli (ST 36). Treatment was performed for 30 minutes every day, 5 days as a course of treatment. There were 2 days off between courses and three courses in total. Differences in a main symptom index of gastroparesis (GCSI) scores, 9 symptom scores, and a health questionnaire (SF-36) were compared between each group before and after treatment and 4 weeks after the end of treatment. The difference of gastric emptying rate was compared before and after treatment.

**Results:**

The GCSI scores of each group after treatment and at follow-up were significantly lower than those before treatment (*P* < 0.01), and the reduction in group A was greater than that of groups B and C (*P* < 0.01). The score of each symptom was meaningfully lower than that before treatment (*P* < 0.01 or *P* < 0.05). The effect was best in group A, followed by group B. After treatment, the barium meal in the stomach of the three groups was significantly reduced compared with before treatment (*P* < 0.01). There was no statistical difference between the groups. The results of SF-36 showed that acupuncture treatment can improve health status, to a certain extent, and there was no significant difference in the three groups.

**Conclusion:**

(1) Acupuncture is an effective method for the treatment of gastroparesis. (2) The combination of Zhongwan (CV 12) with Zusanli (ST 36) showed the most promising effect on relief of the symptoms in patients with gastroparesis. (3) “Selecting acupoints by site” is the key factor affecting the synergy effect of “acupoint compatibility.” This trial was registered with the International Center for Clinical Trials (registration no. NCT02594397).

## 1. Introduction

Gastroparesis refers to a clinical syndrome in which gastric emptying is delayed without mechanical obstruction or other underlying diseases [1, 2], which is characterized by decreased gastric motility, delayed gastric emptying, and gastric rhythm disorder. The main clinical symptoms are loss of appetite, flatulence after meals, heating, nausea, vomiting, abdomen bulge, and diarrhea. The pathogenesis of gastroparesis is closely related to gastric emptying process and also affected by other factors, such as neuropathy, hyperglycemia, gastrointestinal hormone changes, microvascular and gastrointestinal smooth muscle disease, Cajal interstitial cytopathic disease, *Helicobacter pylori* infection, and so on [3]. Studies show that the incidence rate of women is higher than men (37.8/100,000 and 9.6/100,000 for females and males, respectively) [4]. There is no specific treatment for gastroparesis except for the use of gastrointestinal motility drugs, such as metoclopramide, motilin, and cisapride. Although temporary relief of the symptoms can be achieved by using the drugs mentioned above, they cannot cure the disease fundamentally. Moreover, persistent use will result in intolerable side effects, as well as relapse after stopping the drug. According to the literature reports [5–7], modern famous doctors also believe that the compatibility of acupoints is based on the theory of traditional Chinese medicine. Under the guidance of the principle of acupoint selection, a combination of two or more acupoints will produce a synergistic effect, thereby improving clinical efficacy [8]. And acupuncture treatment of gastroparesis can significantly improve clinical efficacy [9].

The basic idea of this study is to take a partial selection point, a distal selection point, and a nonacupoint and then match the “Zusanli (ST 36)” separately, which is divided into three groups, namely, “Zhongwan (CV 12) + Zusanli (ST 36) Group,” “Neiguan (PC 6) + Zusanli (ST 36) Group,” and “Nonacupoint + Zusanli (ST 36) Group.” Three groups of patients were used for the control study to observe the difference in efficacy between Zhongwan (CV 12), Neiguan (PC 6), and nonacupoints. We hope this study will help in the establishment of SOP of acupuncture site selection for the treatment of gastroparesis and promotion of the standardization process of acupuncture so that Chinese medicine and acupuncture can be better transmitted and carried for.

## 2. Materials and Methods

### 2.1. Test Design and Subject Source

The method of a multicenter, randomized, controlled trial design was used in this study. Three clinical centers are the First Affiliated Hospital of Hunan University of Traditional Chinese Medicine, the People's Hospital of Hunan University of Traditional Chinese Medicine, and the Hengyang Hospital affiliated to Hunan University of Traditional Chinese Medicine. The trial protocol has been approved by the Ethics Review Committee of the First Affiliated Hospital of Hunan University of Traditional Chinese Medicine (approval no. HN-LL-KY-2014-004-02) and registered with the International Center for Clinical Trials (registration no. NCT02594397). We employed two strategies to recruit participants with gastroparesis. Firstly, we posted posters in the outpatient department of the hospital and recruited patients who met the inclusion criteria and volunteered to participate in the study. Secondly, the recruitment advertisements were posted on the hospital's website and recruitment posters were distributed in nearby hospitals and communities, which will attract patients to join the study.

### 2.2. Research Methodology

#### 2.2.1. Random Method

The subject adopts a central random method, and the China Academy of Chinese Medical Sciences undertakes the central randomization. The patient information (date of birth, gender) is collected into the central stochastic system of clinical research of China Academy of Chinese Medical Sciences. The patients were assigned to one of Group A, Group B, and Group C by a central stochastic system method. This process ensures that each eligible patient is assigned to any group with the same probability, and the random serial number will not be affected by researchers, operators, evaluators, and statistical analysts.

#### 2.2.2. Blind Method

First, the efficacy evaluation is performed by a third party who does not know the grouping situation. Second, blind statistical analysis is used in the data summary stage to ensure the authenticity of the research results. All CRF data were entered into the Clinical Evaluation Center of China Academy of Chinese Medical Sciences for statistical analysis. Due to the special nature of acupuncture, this study cannot conceal the patient. Therefore, before the treatment, we tell the patient that each person's treatment plan is the best plan according to individual circumstances and explain in detail.

### 2.3. Diagnostic Criteria

According to the diagnostic criteria of the Clinical Management Guidelines for Gastroparesis, the diagnosis of gastroparesis must meet the following three criteria:

1. The patient has symptoms of upper abdominal pain, postmeal fullness, nausea, vomiting, early fullness, and other symptoms of gastroparesis.
2. The patients are not satisfied with the diagnosis of pyloric outlet obstruction caused by pyloric organic lesions.
3. Delayed gastric emptying has been diagnosed.

### 2.4. Inclusion Criteria and Exclusion Criteria

Inclusion criteria: (1) meet the diagnostic criteria for gastroparesis; (2) adult patients under 60 years old (between 18 and 60 years old); (3) those who did not participate in any clinical trials 3 months before admission; (4) female patients who are not pregnant and who are not lactating.

Patients with the following diseases or syndrome are excluded in this trial: (1) patients with reflux esophagitis; (2) patients with postoperative gastroparesis; (3) patients with acute complications such as ketoacidosis and nonketotic hyperosmolar coma; (4) patients with acute cardiovascular and cerebrovascular diseases, severe trauma or surgery, severe infections, and pregnant or lactating women; (5) patients who have had myocardial infarction, acute coronary syndrome, or coronary revascularization; (6) patients with severe hepatobiliary disease or aspartate aminotransferase (AST) and/or alanine aminotransferase (ALT) 2 times higher than the upper limit of normal; (7) patients with kidney damage or serum creatinine exceeding 140umol/L; (8) patients with severe hematopoietic system disease (such as hemoglobin (Hb): male < 110 g/L, female < 100 g/L, white blood cell (WBC) < 3.5 × 109/L, and platelet (PLT) < 80 × 109/L); (9) hypertension (systolic blood pressure ≥ 180 mmHg, diastolic blood pressure ≥ 100 mmHg); (10) progressive malignant tumor or other severely consumptive diseases, susceptible to infection and bleeding; (11) patients with organic lesions such as peptic ulcer found by endoscopy; and (12) those who did not cooperate with the treatment and the disease continued to worsen or had serious complications in the trial.

### 2.5. Withdrawal and Dropout

Withdrawal and dropout are defined as follows:

1. Cases that are not included in the inclusion criteria should be excluded.
2. Subjects with poor compliance, withdrawal during treatment, combined use of the treatment prohibited by this program, or change of treatment on their own.
3. Cases in which serious adverse events or complications have occurred and are not suitable for treatment and have been discontinued.

Principles for treatment of withdrawal and dropout:

1. When the withdrawal or dropout happens, the competent doctor should know the detailed reason, record the last treatment time, and complete the assessment process associated with the subject.
2. The doctor shall treat the patients with an alternative method if the subject withdraws from the clinical trial because of ADR or very poor response.
3. Once the random number is obtained, it becomes the subject of trial observation, regardless of the future diagnosis and whether the treatment is complete.
4. All withdrawal or dropout cases were analyzed for intentionality at the end of the trial.

### 2.6. Suspension of Study Case

1. Patients with adverse events and special physiological changes in the study, which is not suitable for further studies.
2. Patients who have serious complications or worsened their condition during the study and need urgent management.
3. Subjects who withdraw from clinical studies during the trial.
4. Subjects who do not cooperate and who do not obey the plan after repeatedly explained by the clinician.
5. The investigator should record in detail the reason and time of withdrawal from the study. Those who have exceeded 1/2 course of treatment should be included in the efficacy statistics.

## 3. Intervention

### 3.1. Grouping

The study was divided into three groups. The positioning of the acupoints is determined concerning the national standard of the People's Republic of China, “Name and Position of Acupoints” (GB/T 12346-2006) [10].

**Figure 1:**
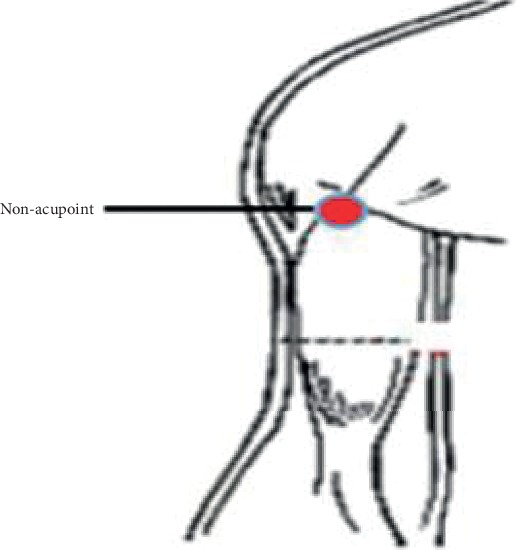
Nonacupoint.

1. Group A: Zhongwan (CV 12) and Zusanli (ST 36); Zhongwan (CV 12) is located in the upper abdomen, on the front midline, 4 inches above the navel; Zusanli (ST 36) is located in the anterior lateral part of the calf, 3 inches below the “Dubi (ST 35),” 1 inch from the anterior edge of the tibia.
2. Group B: Neiguan (PC 6) and Zusanli (ST 36); Neiguan (PC 6) is located in the anterior region of the forearm, 2 inches above the transverse line of the volar, between the palm length tendon and the radial flexor tendon.
3. Group C: nonacupoints and Zusanli (ST 36); nonacupoints are located at the leading edge of the arm, the junction of the deltoid and biceps [11], as shown in Figure 1.

### 3.2. Needle

“Hwato” brand disposable acupuncture needle was produced by Suzhou Medical Products Factory Co., Ltd (production enterprise license: Suzhou Food and Drug Administration production no. 2001-0020; Registration Certificate no. sxzz 20162270970). The specifications are 0.30 mm in diameter and 40 mm long and 0.30 mm in diameter and 50 mm long.

### 3.3. Combination Therapy

During the entire study period, all participants were not allowed to add or subtract any drugs before approval. If the subject has other medical conditions and needs medication, a detailed record of the drug name and dosage should be included in the case report.

### 3.4. Acupuncture Operation

The direction and depth of acupuncture at meridian points strictly followed standards of the “12th Five-Year Plan” textbook “Acupuncture and Moxibustion” of the Ministry of Health which was edited by Liang [12].

1. Group A: acupuncture points include Zhongwan (CV 12) and Zusanli (ST 36). Operation: the patient was supine, and the local skin was wiped with 75% ethanol cotton balls to disinfect. A disposable stainless steel acupuncture needle of 0.30 mm in diameter and 50 mm long was used for the operation. Zhongwan (CV 12): pierce into the depth of 25∼40 mm perpendicularly; Zusanli (ST 36): pierce into the depth of 25∼48 mm perpendicularly.
2. Group B: acupuncture points include Neiguan (PC 6) and Zusanli (ST 36). Operation: the patient was supine, and the local skin was wiped with 75% ethanol cotton balls to disinfect. Neiguan (PC 6): A disposable stainless steel acupuncture needle of 0.30 mm in diameter and 40 mm long was used, and the needle was inserted perpendicularly into the skin with a depth of 15–25 mm; Zusanli (ST 36): A disposable stainless steel acupuncture needle of 0.30 mm in diameter and 50 mm long was used, and the needle was inserted perpendicularly into the skin with a depth of 25–48 mm.
3. Group C: acupuncture points include nonacupoint and Zusanli (ST 36). Operation: the patient was supine, and the local skin was wiped with 75% ethanol cotton balls to disinfect. A disposable stainless steel acupuncture needle of 0.30 mm in diameter and 50 mm long was used for the operation. Nonacupoint: pierce into the depth of 30∼40 mm perpendicularly; Zusanli (ST 36): pierce into the depth of 25∼48 mm perpendicularly.

Needle adjustment: “twirling method” and “lifting-thrusting methods” were performed after the needle is inserted to get the needle sensation. The amplitude of the “twirling method” was between 0.3 and 0.5 cm, and the frequency was between 60 and 90 times/min; the angle of the “lifting-thrusting methods” was between 90 and 180 degrees, and the frequency was between 60 and 90 times/min. Needles were maintained on the human body for 30 min with each treatment. Needle was adjusted every 10 minutes and lasted for 1 minute each time. Treatment was performed for 30 minutes every day, 5 days as a course of treatment. There were 2 days off between courses and three courses in total.

## 4. Observation Index

### 4.1. Main Efficacy Index

The total score of the gastroparesis cardinal symptom index (GCSI) was used as the main efficacy index of the study. GCSI can be further divided into 9 subitems, which are early fullness, stomach bloating, feeling of fullness after a meal, nausea, vomiting, loss of appetite, eating less, flatulence, and abdominal bulge. Each subitem has 6 rating levels: none, very slight, mild, moderate, severe, and very serious, with a score of 0-5. The higher the score, the more severe the symptoms. GCSI and 9 subitems were scored at week 0, 3, and 7. Statistical analysis was performed on pretreatment, posttreatment, and follow-up data. The overall efficacy standard was determined by the Cochrane Multicenter Clinical Trial Coordination Group in China [13]. The efficacy index was obtained according to the following formula: efficacy index = ((pretreatment total score or a total score of follow-up period − total score after treatment) ÷ total score before treatment) × 100%. Significant effect: efficacy index ≥ 75%; effective: 25% ≤ efficacy index < 75%; invalid: efficacy index < 25%.

### 4.2. Secondary Efficacy Index

1. Detection of gastric emptying rate by the X-ray method [14]: patients are advised to fast after 19 : 00 on the night before the experiment and water is banned after 24 : 00. Patients can take the standard meal at 7 : 00 on the day of the experiment: 200 ml of water, 80 g of instant noodles, 50 g of sausage, and special capsules (20 purlins, 4/grain, a total of 5 capsules), and they can eat evenly within 15 minutes. Patients can stand, walk, and sit after a meal, but they cannot sleep. The abdominal plain film was taken at the 4th hour after the meal, and the number of remaining barium meal in the stomach area of the abdominal plain film was calculated after 4 hours. If the location is unclear, the patient may be given a gas powder (about 1.5 grams) or a perspective position. The emptying rate can be calculated as follows: emptying rate = (20 − number of stomach barium meals)/20 × 100%. The barium meal X-ray was detected before treatment and after treatment.
2. Health Survey Summary (SF-36): the scoring criteria are divided into 8 dimensions—physiological function (PF), role physical (RP), bodily pain (BP), general health (GH), vitality (VT), social function (SF), role emotional (RE), and mental health (MH), and they include 36 scoring entries. After scoring by each dimension scoring method, the conversion scores of each dimension are calculated by the range method, and the total score is the sum of the conversion points of each dimension. The higher the score, the better the health status. SF-36 was recorded at weeks 0, 3, and 7.

### 4.3. Observation Period

The observation period for this trial was 8 weeks (from week 0 to 7), including the baseline period (1 week), treatment period (3 weeks), and follow-up period. Treatment was performed for 30 minutes every day, 5 days as a course of treatment. There were 2 days off between courses and three courses in total. A follow-up was performed 4 weeks after the end of treatment.

### 4.4. Safety Assessment

Before randomization, all participants were required to measure blood routine, stool routine, urinary routine, liver and kidney function, electrocardiogram, and self-rating measures of anxiety and depression. These tests will help identify and exclude participants with heart, liver, and kidney disorders or other serious illnesses.

Adverse events are defined as any signs, symptoms, or illnesses that are unexpected or uncomfortable. These adverse events include bleeding, hematoma, fainting, severe pain, and local infections caused by acupuncture. Any adverse events and details that occur throughout the observation period should be documented.

During the observation period, if the patient has serious adverse reactions, or the subject voluntarily withdraws from the clinical study, it is not appropriate to continue to participate in the study. The investigator must find out the cause and record it in the case report form. Patient compliance was also recorded at the end of treatment and during the follow-up period.

## 5. Statistical Methods

Statistical analysis of the data was performed using SAS 9.3 statistical analysis software. The measurement data of each visit point were described statistically using the mean ± standard deviation. Measurement data that obeyed the normal distribution before and after treatment in the group were tested by paired *t*-test, while those that did not obey the normal distribution were tested by the Wilcoxon test. Measurement data between groups were tested by ANOVA or nonparametric tests. The count data of adverse events were described by frequency (composition ratio), and the incidence of adverse events between the two groups was compared by the chi-square test. All statistical tests are two-sided, and *P* ≤ 0.05 is considered statistically significant.

## 6. Results and Analysis

### 6.1. Medical Record Enrollment

A total of 99 patients with gastroparesis were included in the study. Two cases were shed during the study. The rate of shedding was 2.02% < (10%). As shown in Table 1, the completion of the experiment was 97 cases, of which 33 cases were in group A, 32 cases in group B, and 32 cases in group C. There were no significant differences in the general information, such as gender, age, course, height, weight, whether they had received acupuncture treatment, complications, and the evaluation of acupuncture expectation values (*P* > 0.05), indicating that the data were comparable, as shown in Table 2.

### 6.2. Comparison of GCSI

**Figure 2:**
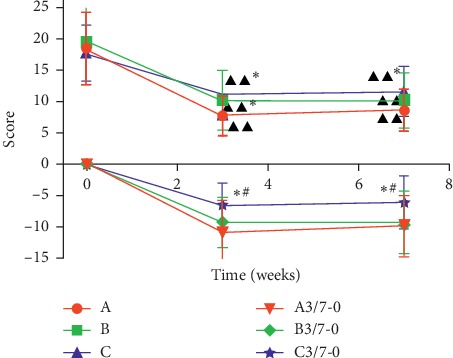
Comparison of GCSI scores. 3-0 and 7-0 are the scores obtained by the 3rd and 7th weekend scores minus the 0th week. Data were expressed as mean ± *s* (*n* = 33). ^*∗*^*P* < 0.05, compared with Zhongwan (CV 12) + Zusanli (ST 36) group; ^#^*P* < 0.05, compared with Neiguan (PC 6) + Zusanli (ST 36) group; ^ΔΔ^*P* < 0.01, compared with baseline period.

There were no significant differences in GCSI scores at baseline between groups. Compared with the baseline period, the scores of the three groups after treatment (3rd weekend) and follow-up (7th weekend) were significantly lower (*P* < 0.01). Comparing the differences in efficacy between the groups, the results showed that the short-term efficacy (3rd weekend) and long-term efficacy (7th weekend) of group A and group B were the best (*P* < 0.05), as shown in Figure 2. It shows that the symptoms of patients after acupuncture treatment were significantly relieved. After treatment, the effective rates of A, B, and C groups were 48.48%, 30.30%, and 12.12%, respectively, as shown in Table 3. The effective rates of A, B, and C groups during the follow-up periods were 93.94%, 90.91%, and 78.79%, respectively, as shown in Table 4.

### 6.3. Comparison of 9 Symptom Scores in Patients with Gastroparesis

**Figure 3:**
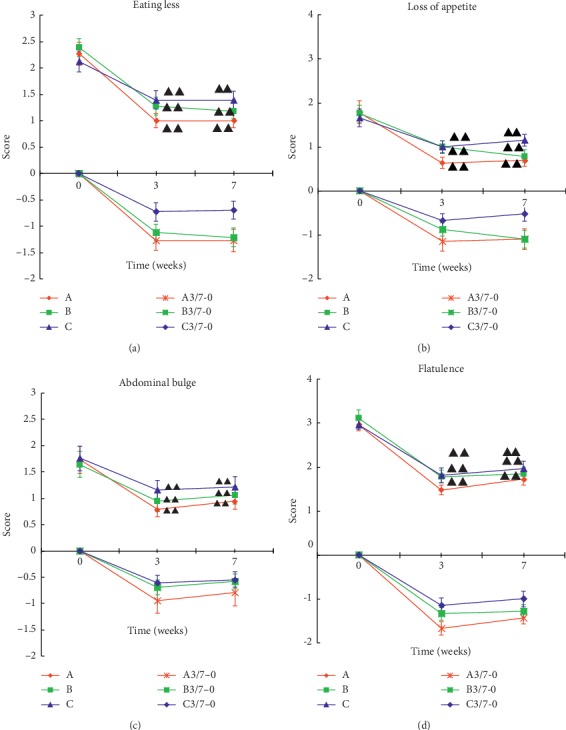
Comparison of four symptom scores in each group of patients. 3-0 and 7-0 are the scores obtained by the 3rd and 7th weekend scores minus the 0th week. Data were expressed as mean ± *M* (*n* = 33). ^ΔΔ^*P* < 0.01, compared to baseline.

**Figure 4:**
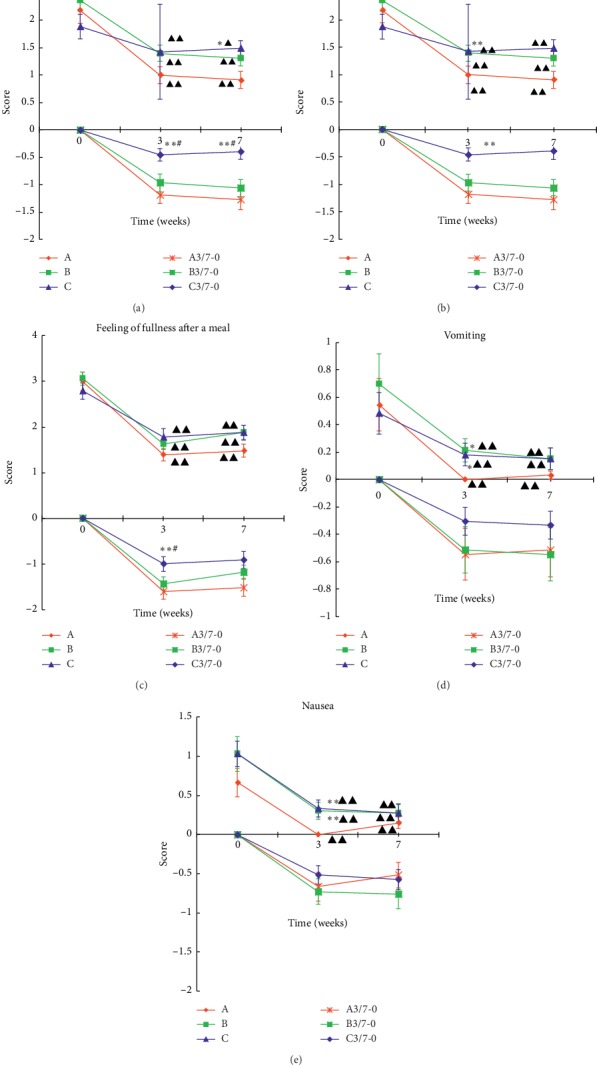
Comparison of five symptom scores in each group. 3-0 and 7-0 are the scores obtained by the 3rd and 7th weekend scores minus the 0th week. Data were expressed by mean ± *M* (*n* = 33). ^*∗*^*P* < 0.05, ^*∗∗*^*P* < 0.01, compared with Zhongwan (CV 12) + Zusanli (ST 36) group; ^#^*P* < 0.05, ^##^*P* < 0.01, Neiguan (PC 6) + Zusanli (ST 36) group comparison; ^ΔΔ^*P* < 0.01, compared with the baseline period.

The results of the comparison before and after treatment showed that the nine symptom scores of the three groups after treatment and follow-up period were significantly lower than the baseline period (*P* < 0.01), as shown in Figures 3 and 4. The results of the comparison of the efficacy differences between the three groups showed that there was a significant difference in the scores of the effects of acupuncture on “early fullness, stomach bloating, and feeling of fullness after a meal,” while the efficacy scores of “eating less, loss of appetite, flatulence, abdominal bulge, nausea, and vomiting” were not significantly different between the three groups (*P* > 0.05). The results of the “early fullness” data show the following (Figure 4(a)): at the third weekend, the effects of the A and B groups were more significant than those of the C group (*P* < 0.01 or *P* < 0.05). The efficacy of the A and B groups was still better than that of the C group (*P* < 0.01) at the seventh weekend. The results of the “stomach bloating” data show the following (Figure 4(b)): at the third weekend, the efficacy of group A was significantly better than that of group C (*P* < 0.01). There was no significant difference between the groups at the seventh weekend (*P* > 0.05). The results of “feeling of fullness after a meal” data show the following (Figure 4(c)): at the third weekend, the effects of group A and group B were more significant than those of group C (*P* < 0.01 or *P* < 0.05). There was no significant difference in efficacy between the groups at the seventh weekend (*P* > 0.05).

### 6.4. Comparison of Gastric Emptying

**Figure 5:**
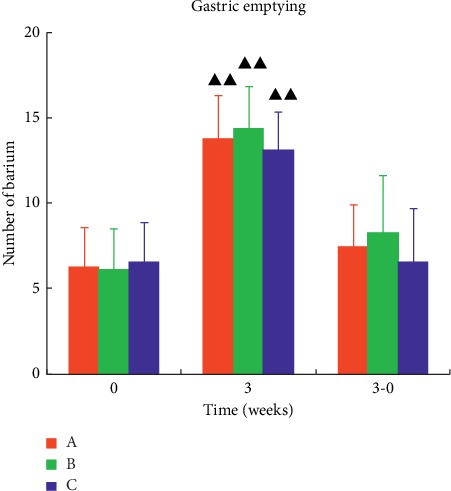
Comparison of gastric emptying before and after treatment in each group of patients. The value is “20—the number of remaining bars,” and the data were expressed as mean ± *s* (*n* = 33). ^ΔΔ^*P* < 0.01, compared with the baseline period.

The amount of barium meal in each group was significantly lower than that in the previous treatment (*P* < 0.01). But there was no significant difference between the three groups. It shows that acupuncture treatment has a significant effect on promoting gastric emptying, which is worthy of clinical application. In the study, the three groups were equally effective in promoting gastric emptying, as shown in Figure 5.

### 6.5. Comparison of Health Survey Scores

**Figure 6:**
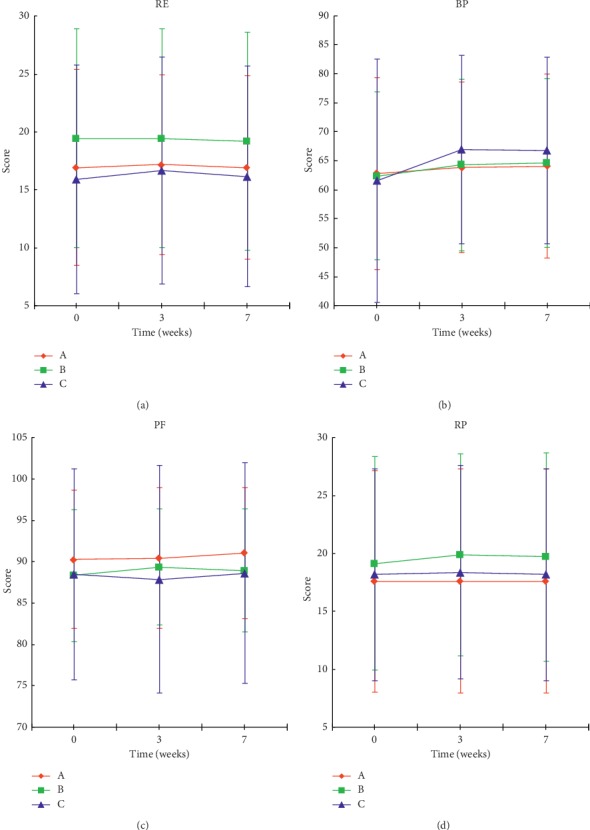
Comparison of the scores of the four health survey dimensions of each group of patients. The data were expressed as mean ± *s* (*n* = 33).

**Figure 7:**
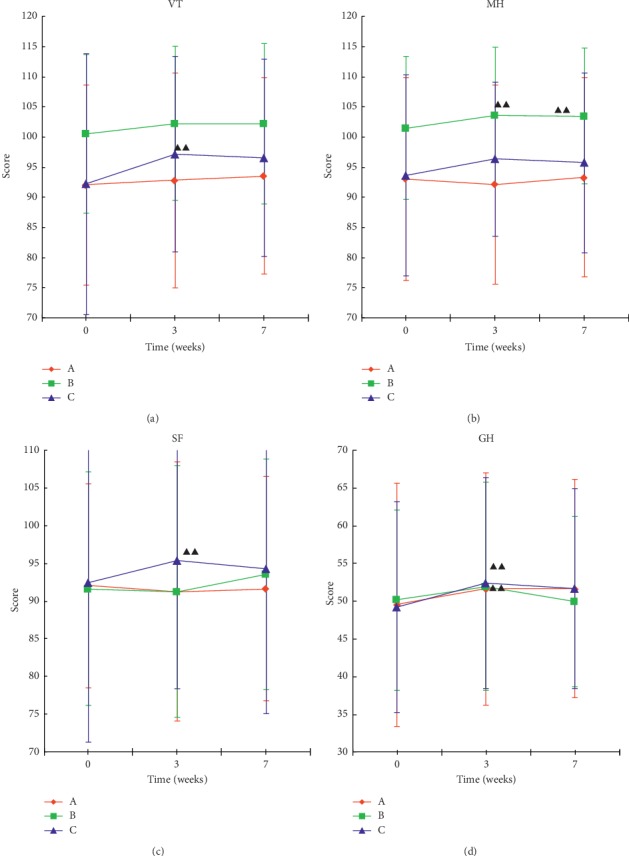
Comparison of the scores of the four health survey dimensions of each group of patients. Data were expressed as mean ± *s* (*n* = 33). ^ΔΔ^*P* < 0.01, compared to baseline.

The health surveys were scored from 8 dimensions, and there was no statistical difference between the three groups for each dimension, indicating that there was no significant difference in the three groups between the health survey scores. Comparisons before and after treatment showed that the “PF, RP, BP, and RE” did not differ significantly before and after treatment (Figure 6). The “VT, MH, SF, and GH” showed a certain effect after treatment (Figure 7). The comparison of VT (Figure 7(a)) showed that the scores of group C at the third weekend were significantly higher than those before treatment (*P* < 0.01), while the scores of group A and B were not significantly different than those before treatment (*P* > 0.05). A comparison of MH (Figure 7(b)) showed that the scores of group B after treatment and follow-up period were significantly higher than those before treatment (*P* < 0.01), and there was no significant change in the other groups. Comparison of SF (Figure 7(c)) showed that the scores of group C at the third weekend were significantly higher than those before treatment, and there were no significant changes in the other groups. A comparison of GH (Figure 7(d)) showed that the scores of groups A and C were significantly higher than those before the treatment. The results showed that acupuncture treatment can improve health status to a certain extent.

### 6.6. Safety Evaluation

#### 6.6.1. Adverse Event

There were no significant differences between the groups in the occurrence of adverse events during the trial, as shown in Table 5.

#### 6.6.2. Laboratory Examination

Each group of patients in this study underwent a laboratory examination before and after treatment, including blood routine, urine routine, stool routine, liver function, and renal function. No heart, liver, and kidney disorders or other serious illnesses were found.

## 7. Discussion

This study was conducted in 3 clinical trial centers by adopting a randomized, controlled trial design, and the results confirmed that the clinical efficacy of “selecting acupoints by site” for treating gastroparesis is certain. A large number of subjects said that their symptoms improved after treatment. After treatment and follow-up, the GCSI score and 9 symptom scores of group A, group B, and group C were significantly lower than those before treatment, and the efficacy of group A and group B was better than that of group C. In terms of the gastric emptying rate, the number of barium meal in each group was significantly reduced after treatment. There was no significant difference between the three groups, indicating that acupuncture treatment has an obvious curative effect on the gastric emptying rate, and the three groups have similar effects in promoting gastric emptying. The results of SF-36 showed that acupuncture treatment can improve the health status to a certain extent.

The ideas of this study are to study the factors affecting the synergy effect of “acupoint compatibility,” which is different from previous studies. Most of the previous studies are about the influencing factors of “acupuncture efficacy,” including stimulation methods, length of time, and body function. However, the main factors affecting the synergy effect of “acupoint compatibility” are acupoint selection and acupoint characteristics. The method of selecting acupoints is divided into “site-based compatibility” and “meridian-based compatibility.” “Selecting points by syndrome differentiation” and “selecting points based on the clinical experience” are the basic requirements for acupoint compatibility [15]. According to the analysis of acupuncture treatment of gastroparesis, the top three acupoints with the highest frequency of use in all acupoints are Zhongwan (CV 12), Zusanli (ST 36), and Neiguan (PC 6) [16]. “Zhongwan (CV 12)” is the acupoint on “Ren meridian,” which is the Front Mu Point of the stomach meridian (ST). It is located in the upper abdomen and the stomach is next to the body surface projection. Stimulation of the acupoint can directly affect the gastrointestinal tract and regulate the function of the gastrointestinal tract. “Zusanli (ST 36)” is an acupuncture point on stomach meridian (ST), which is the He-Sea acupoint and Lower He-Sea acupoint of stomach meridian (ST). Therefore, Zusanli (ST 36) is a common acupuncture point for the clinical treatment of gastrointestinal diseases. “Neiguan (PC 6)” is the acupuncture point on the pericardium meridian (PC), which are not only the Luo-Connective acupoints but also the Eight Confluence Points and the Intersection Points. It is located at the distal end of the upper extremity and can be used for the treatment of gastrointestinal diseases such as stomach pain, vomiting, and hiccups. The selection of this point is both a specific acupoint selection point and a distal selection point.

Gastroparesis is characterized by reduced contractility of smooth muscle, weakened gastric motility, no tension, emptying delay of the antrum, and prolonged pyloric contraction. Clinical manifestations include loss of appetite, nausea, paroxysmal vomiting, abdominal discomfort, and abdominal distension. A large number of modern clinical trials have proved that acupuncture is effective in the treatment of gastroparesis [17, 18]. In recent years, the clinical studies of gastroparesis are mostly compared with the acupuncture group and the modern medicine group. The efficacy of each study is not the same and the difference may due to factors such as “selection of acupoints, number of acupuncture points, stimulation means and degree, time, and body state of different patients.” In general, the selection of points in different studies is somehow ambiguous, and there is no uniform standard for compatibility, which is already a big obstacle for optimizing the therapeutic effect of acupuncture therapy. Zhu Bing's studies show that stimulating local acupoints in the abdomen can inhibit bowel movement while stimulating distal acupoints can promote bowel movement [19, 20]. Therefore, acupuncture treatment of gastroparesis has important significance and shows a very promising future [21].

In summary, this experiment aims to explore the key factors affecting the synergy effect of “acupoint compatibility.” The patients with gastroparesis were divided into three groups according to the acupoint combination, namely, Zusanli (ST 36) acupoint plus the local acupoint (Zhongwan (CV 12)), Zusanli (ST 36) acupoint plus the distal acupoint Neiguan (PC 6), and Zusanli (ST 36) acupoint plus the nonacupoint. The GCSI, gastric emptying rates, and SF-36 were used as indicators for comparative observation. According to the research results, the preliminary conclusions can be drawn as follows: (1) acupuncture is an effective method for the treatment of gastroparesis; (2) Zusanli (ST 36) plus the local acupoint (Zhongwan (CV 12)) has the best effect in improving the symptoms of gastroparesis in patients, followed by Zusanli (ST 36) plus the distal acupoint (Neiguan (PC 6)), which is better than Zusanli (ST 36) plus the nonacupoint; (3) “selecting acupoints by site” is the key factor affecting the synergy effect of “acupoint compatibility.”

## Figures and Tables

**Table 1:** Pathological enrollment.

	Enrollment				FAS/SS				PPS			
	A	B	C	Total	A	B	C	Total	A	B	C	Total
The First Affiliated Hospital of Hunan University of Traditional Chinese Medicine	11	11	11	33	11	11	11	33	11	11	11	33
Hengyang Hospital affiliated to Hunan University of Traditional Chinese Medicine	19	19	19	57	19	19	19	57	19	18	18	55
People's Hospital of Hunan University of Traditional Chinese Medicine	3	3	3	9	3	3	3	9	3	3	3	9
Total	33	33	33	99	33	33	33	99	33	32	32	97

*Definition of abbreviations*: FAS = full-text analysis set; SS = safety set; PPS = per-protocol set.

**Table 2:** Comparison of baseline data.

Group	Age (year)	Gender		The course of the disease (month)			Height (cm)	Body weight (kg)	Accepted or not treated with acupuncture (case)		Combined or not combined with the disease (case)	
		Men	Women	Max	min	mean($\bar{x}$±*s*)			Yes	No	Yes	No
A	47.88 ± 13.20	22	11	190	12	58.76 ± 38.96	163.36 ± 8.22	56.81 ± 7.54	9	24	9	24
B	49.94 ± 12.05	24	9	207	2	68.64 ± 51.92	160.55 ± 6.32	57.16 ± 8.00	11	22	11	22
C	43.18 ± 15.03	23	10	159	8	49.82 ± 29.75	160.76 ± 7.75	56.05 ± 7.47	6	27	6	27
*P* value	0.1188	0.87	0.1845	0.2384	0.8335	0.37	0.37					

Data are presented as mean ± SD (*n* = 33).

**Table 3:** Overall efficacy evaluation of patients in each group after treatment (3rd weekend).

Group	Significant effect	Effective	Invalid	Efficient(%)
A	0	16	17	48.48
B	1	9	23	30.30
C	1	3	29	12.12

**Table 4:** Overall efficacy evaluation of patients in each group during the follow-up period (7th weekend).

Group	Significant effect	Effective	Invalid	Efficient (%)
A	3	28	2	93.94
B	2	28	3	90.91
C	1	25	7	78.79

**Table 5:** Adverse events.

	A	B	C	Total	*P* value
No	26 (78.79)	28 (84.85)	31 (93.94)	85	0.2434
Yes	7 (21.21)	5 (15.15)	2 (6.06)	14	
Total	33	33	33	99	

## Data Availability

All CRF data were entered into the Clinical Evaluation Center of China Academy of Chinese Medical Sciences, and statistical analysis was performed by a third party (website: http://www.tcmcec.net/). The data used to support the findings of the study are available from the corresponding author upon request.
